# MAFLD: an optimal framework for understanding liver cancer phenotypes

**DOI:** 10.1007/s00535-023-02021-7

**Published:** 2023-07-20

**Authors:** Harry Crane, Cameron Gofton, Ankur Sharma, Jacob George

**Affiliations:** 1 Storr Liver Centre, Westmead Institute for Medical Research, Westmead Hospital and University of Sydney, Sydney, New South Wales Australia; 2 Department of Gastroenterology and Hepatology, Royal North Shore Hospital, 1 Reserve Road, St Leonards, New South Wales Australia; 3 Harry Perkins Institute of Medical Research, QEII Medical Centre and Centre for Medical Research, 6 Verdun Street, Nedlands, Perth, WA 6009 Australia; 4 Curtin Medical School, Curtin Health Innovation Research Institute (CHIRI), Curtin University, Perth, WA 6102 Australia

**Keywords:** MAFLD, Hepatocellular carcinoma, Dual-aetiology, Epidemiology, Screening

## Abstract

Hepatocellular carcinoma has a substantial global mortality burden which is rising despite advancements in tackling the traditional viral risk factors. Metabolic (dysfunction) associated fatty liver disease (MAFLD) is the most prevalent liver disease, increasing in parallel with the epidemics of obesity, diabetes and systemic metabolic dysregulation. MAFLD is a major factor behind this sustained rise in HCC incidence, both as a single disease entity and often via synergistic interactions with other liver diseases. Mechanisms behind MAFLD-related HCC are complex but is crucially underpinned by systemic metabolic dysregulation with variable contributions from interacting disease modifiers related to environment, genetics, dysbiosis and immune dysregulation. MAFLD-related HCC has a distinct clinical presentation, most notably its common occurrence in non-cirrhotic liver disease. This is just one of several major challenges to effective surveillance programmes. The response of MAFLD-related HCC to immune-checkpoint therapy is currently controversial, and is further complicated by the high prevalence of MAFLD in individuals with HCC from viral aetiologies. In this review, we highlight the current data on epidemiology, clinical characteristics, outcomes and screening controversies. In addition, concepts that have arisen because of the MAFLD paradigm such as HCC in MAFLD/NAFLD non-overlapping groups, dual aetiology tumours and MAFLD sub-phenotypes is reviewed.

## Introduction

Hepatocellular carcinoma (HCC) is a major global public health challenge. Already the third leading cause of cancer-related mortality, deaths attributable to HCC are predicted to grow at a rate exceeding that of all other commonly encountered cancers, from 800,000 in 2020 to 1.3 million by 2040 [1]. Global efforts to tackle the traditional risk factors such as hepatitis B virus (HBV) and hepatitis C virus (HCV) have been counteracted by a rise in fatty liver disease driven by epidemics of obesity, type 2 diabetes mellitus (T2DM) and metabolic dysfunction. Metabolic (dysfunction) associated fatty liver disease (MAFLD) is now a substantial contributor to the global HCC burden [2], either as the primary aetiology of liver disease or in combination with other aetiologies such as hepatitis B and hepatitis C virus (HBV and HCV) infection, and alcohol-related liver disease (ARLD).

**Fig. 1:**
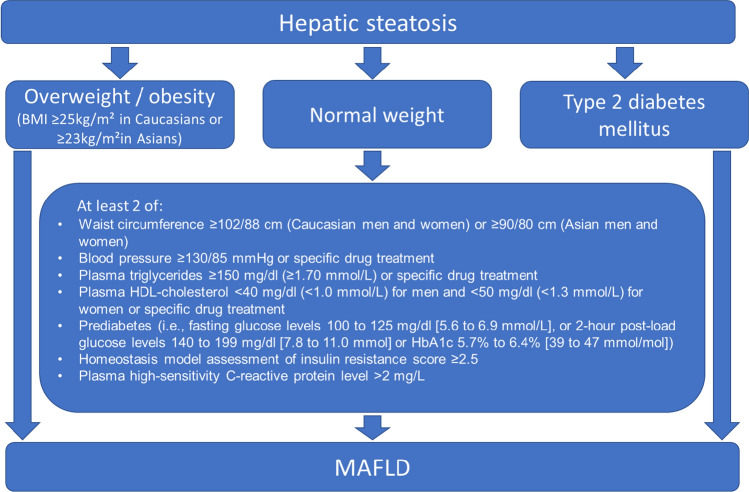
MAFLD diagnostic algorithm (adapted with permission)

In response to the global disease burden of fatty liver disease, an international panel of experts undertook a revision of the nomenclature and diagnostic criteria to align better with the current understanding of the disease as the hepatic manifestation of systemic metabolic dysregulation. Thus, in 2019, the previous term non-alcoholic fatty liver disease (NAFLD) was proposed to be replaced by MAFLD [3]. HCC categorisation using terminology such as “non-viral” or “non-B non-C” is also replaced by MAFLD [4]. Importantly, exclusion of alcohol, viruses, or other causes of steatosis is no longer required for diagnosis, rather the diagnosis can be made “positively” in the presence of steatosis with evidence of metabolic dysfunction, defined by the presence of one of the following three criteria: (1) overweight/obesity (2) T2DM, or (3) evidence of metabolic dysregulation. The latter is defined by the presence of at least two metabolic risk abnormalities listed in Fig. 1 [5, 6]. These criteria give rise to distinct but overlapping clinical sub-phenotypes, namely overweight/obese MAFLD, MAFLD with T2DM and MAFLD in individuals of normal weight.

The aim of this review is to provide an update on MAFLD-associated HCC, including epidemiology, mechanisms, challenges in screening, clinical characteristics and outcomes. In addition, concepts that have arisen from MAFLD such as HCC in MAFLD/NAFLD non-overlapping groups, dual aetiology cancers and MAFLD sub-phenotypes will be reviewed.

## MAFLD, NAFLD, non-overlapping groups

**Fig. 2:**
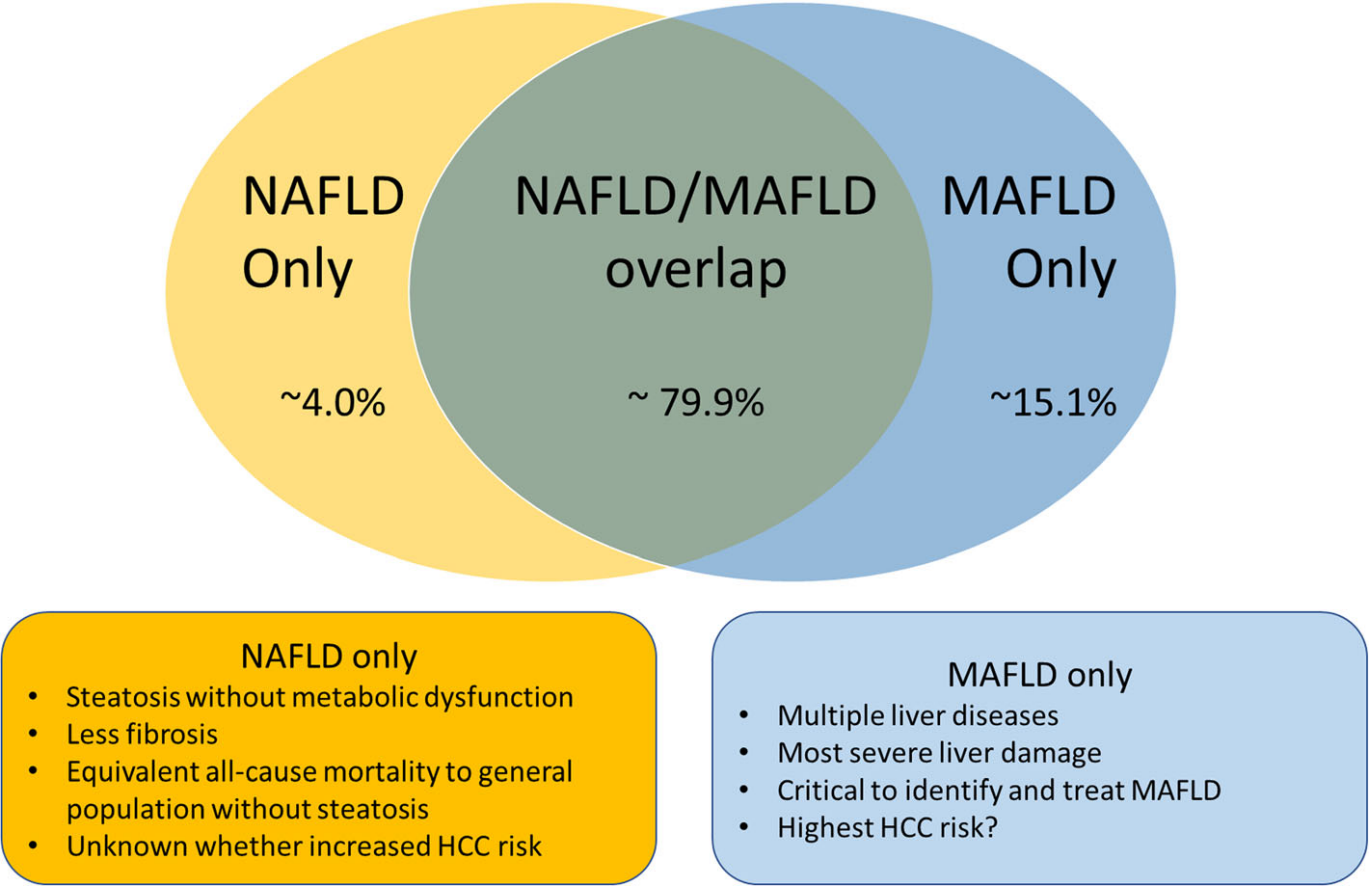
NAFLD/MAFLD overlap

Although there is considerable overlap between NAFLD and MAFLD disease definitions, the terms are not interchangeable, in that there are non-negligible proportions of individuals who have NAFLD but not MAFLD and vice versa (Fig. 2**)**. A meta-analysis comprising 9,808,677 patients estimated 79.9% of patients with fatty liver disease met both disease definitions, 4.0% had NAFLD-only and 15.1% had MAFLD-only [7]. Patients with MAFLD-only are characterised by the presence of steatosis and metabolic dysfunction, with a secondary cause of steatosis which excludes them from a NAFLD diagnosis (such as viral hepatitis, excessive alcohol consumption or medications). The same meta-analysis reported that these patients have the highest prevalence of fibrosis (as measured by elastography or FIB-4 > 2.67) of 10.2% (vs 4.9% in the overlap group, 3.2% in patients without steatosis, and 2.2% in NAFLD only group), as well as higher ALT and AST compared to NAFLD. As the MAFLD concept is still in its infancy, the HCC incidence of this group has not yet been well defined, however, MAFLD-only have one (or multiple) superimposed liver diseases with a propensity for more severe liver damage. Thus, it is essential that all liver diseases be identified so that they can be managed accordingly.

In contrast, patients with NAFLD-only are characterised by hepatic steatosis with normal body weight, absence of T2DM and < 2 markers of metabolic dysfunction. Whether these patients have an increased risk of HCC is not established, however, it should be noted that many studies have shown this subgroup of patients to have the lowest rates of advanced fibrosis and equivalent all-cause mortality risk to patients with no steatosis [8–10]. One Taiwanese insurance registry study did report an increased HCC risk in a cohort of patients with “NAFLD without metabolic syndrome” (albeit not synonymous to NAFLD without MAFLD) relative to individuals without steatosis, however, this cohort may have included a significant portion of patients who did in fact have MAFLD due to the different disease definition used. That study also confirmed a strong link between the extent of metabolic dysregulation and HCC risk with T2DM being the most significant individual risk factor [11]. Another retrospective study of 1286 patients who underwent liver biopsy at an Italian centre reported that HCC did occur in a NAFLD-only cohort in the absence of metabolic dysfunction (defined using the MAFLD definition, albeit without waist circumference or CRP data), but who had genetic predisposition with high-risk alleles of PNPLA3 and TM6SF2. The authors concluding that MAFLD may miss genetically predisposed individuals who do not have metabolic dysfunction [12]. However, conflicting with this are several studies that suggest metabolic dysfunction is a prerequisite for adverse liver outcomes in genetically predisposed individuals. A large study from UK biobank participants reported that while genetic variants amplify HCC risk in patients with metabolic dysfunction, no significant increase in HCC risk in metabolically healthy patients was reported [13]. A Chinese study also reported that in patients with PNPLA3/TM6SF2 variants, metabolic dysfunction was a prerequisite for the development of steatosis [14]. Another study similarly found low rates of hepatic steatosis in PNPLA3 p.I148M variants in individuals of normal bodyweight. Together, these results suggest genetic variants might act more as an amplifier or disease modifier than a disease driver [15]. Further research on HCC risk in individuals with NAFLD without MAFLD is warranted, especially amongst those with a genetic predisposition.

## MAFLD HCC epidemiology

The obesity epidemic has been well documented, fuelled by a global behavioural trend towards increased caloric intake, poor diet quality, a reduction in energy expenditure and an ageing population [16]. The result has been a six-fold increase in the global prevalence of obesity since 1975 [17], and has led to MAFLD becoming the most prevalent chronic liver disease [18]. A recent meta-analysis estimated the overall prevalence of MAFLD to be 38.77% [19]. Globally, overweight / obesity (BMI ≥ 25 kg/m^2^) prevalence is predicted to rise from 2.6 billion to over 4 billion people by 2035, constituting more than 50% of the global population. Obesity (BMI ≥ 30 kg/m^2^) prevalence will rise from 14 to 24% over the same period [20]. Given the prevalence of MAFLD among overweight/obese adults is 50.7% [21, 22], the MAFLD epidemic shows no signs of abating.

Multiple studies have evaluated the risk of HCC development in NAFLD cohorts, with yearly HCC incidence between 0.9 and 2.6% in western cohorts amongst individuals with cirrhosis [2, 23, 24]. Approximately 38% of HCC in patients with NAFLD occurs in individuals without cirrhosis [25], however, annual incidence rates are substantially lower with rates of 0.1 to 1.3 per 1,000 patient years reported [26, 27]. MAFLD tends to be associated with worse markers of liver damage and fibrosis as well as more metabolic comorbidity compared to NAFLD [28, 29], all factors associated with elevated HCC risk in non-cirrhotic MAFLD [30]. Despite this, no studies have directly compared HCC incidence between the different definitions.

MALFD-related HCC is increasing as a proportion of total HCC. An Italian study using the large ITA.LI.CA registry reported that patients with MAFLD as a single disease aetiology had increased as a proportion of total HCC from 3.6% in 2002/2003 to 28.9% in 2018/2019. The proportion of HCV-related HCC decreased over this time (64.4% to 45.8%) as did HBV (15.7% to 10.6%) and alcohol (14.5% to 12.4%). MALFD was modelled to overtake HCV as the single greatest cause of HCC in 4–6 years. Remarkably, in 10–12 years, the investigators predicted that virtually all HCC in Italy would be either MAFLD, or “mixed MAFLD”[31]. Another Swiss study utilised Geneva Cancer Registry data and reported that the proportion of HCC attributable to MAFLD increased from 21% between 1990 and 1994 to 68% from 2010 to 2014, while NAFLD/MAFLD as a single aetiology increase from 2 to 12% in men and from 0 to 29% in women over the same period [32]. Similarly in Japan, MAFLD as a single disease aetiology increased five-fold from 1.5% pre-2008 to 7.2% post-2014 [33].

While MAFLD is traditionally thought of as a Western disease, this not the case. The prevalence of MAFLD in Asia and Middle East and North Africa (MENA) region has been increasing since the 1990s in line with rising rates of obesity and metabolic syndrome. In fact, Asian and MENA countries have experienced a steep rise in liver-related deaths attributable to MAFLD in recent decades, and these regions now account for a larger proportion of deaths than European and American populations [34, 35]. HBV and HCV remain the predominant risk factors for HCC in Asia, but with improving vaccination and treatment programs, between 2006 and 2019, there has been a decline in the incidence rate for HCC owing to HBV (4.08 to 3.81 per 100,000) and HCV (2.65 to 2.17 per 100,000), while HCC owing to MAFLD increased (0.48 to 0.50 per 100,000) over the same period [36]. MALFD is likely to continue to offset the gains made by the reducing incidence of viral HCC in the future.

A distinct subgroup of MAFLD which has generated increasing interest and challenges our understanding of MAFLD pathogenesis is so called “lean-MAFLD”, that is individuals who develop MAFLD within a normal BMI category (BMI 18.5–24.9 kg/m_2_ among those of European descent, or 18.5–22.9 kg/m_2_ in Asian populations). A meta-analysis of 93 studies reported a prevalence of lean MAFLD in the global MAFLD population of 19.2%, constituting 5.1% of the general population [37]. Interestingly, several studies have reported worse long-term liver outcomes in lean—as compared to obese-MAFLD including more severe fibrosis, higher rates of progression to severe liver disease and transplantation [38–40]. Regarding HCC risk, few studies have compared HCC incidence between lean-MAFLD and obese-MAFLD, however, the available data would suggest the rates are similar [41, 42]. Of note, the ITA.LI.CA registry study reported 32.26% of patients with MAFLD HCC had the lean-MAFLD phenotype, while a strikingly high proportion (52.81%) of patients with HCC from another aetiology met the definition of lean-MAFLD.

## Combined aetiology HCC

**Fig. 3:**
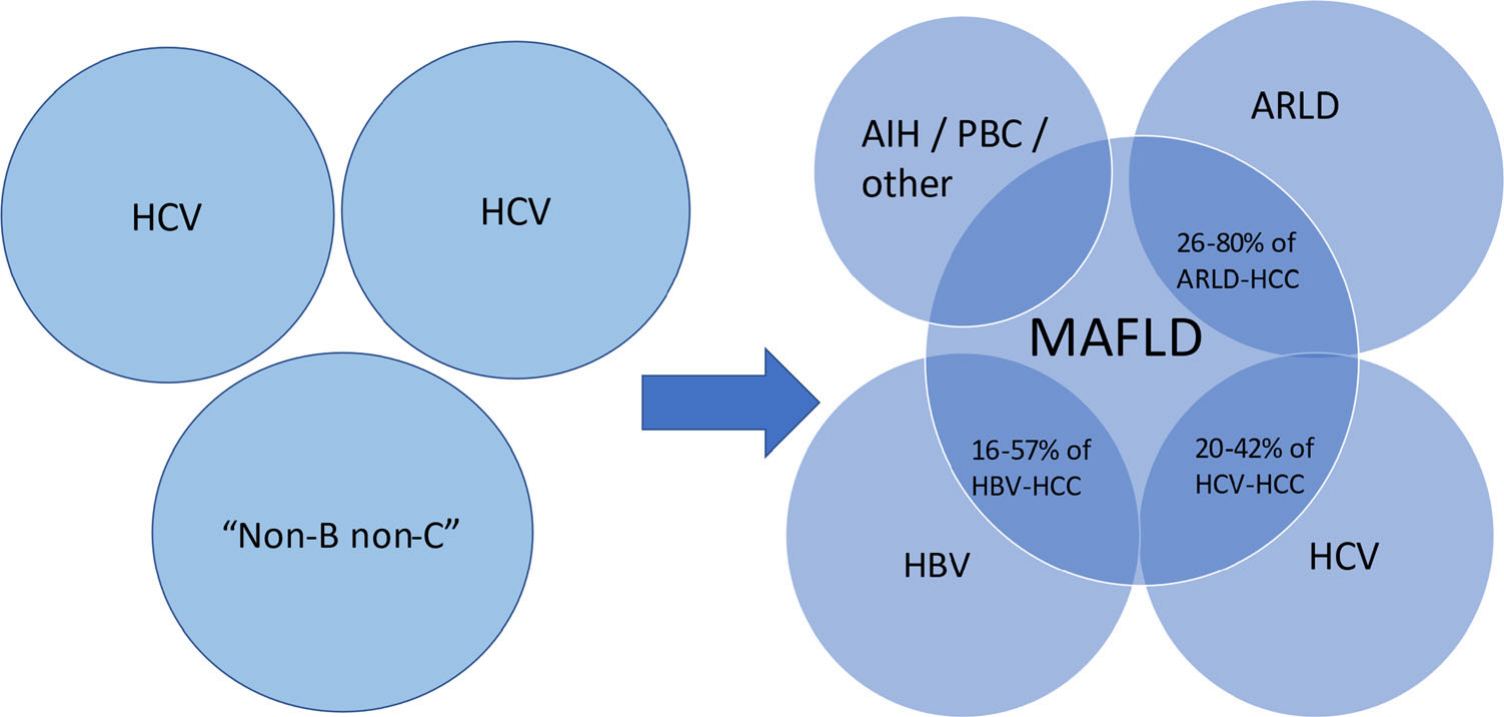
Old paradigm of attributing HCC to a single aetiology. In reality, there is substantial overlap between MAFLD-HCC with other liver diseases, yet little is known about the distinct mechanisms, outcomes and response to treatments in these “dual-aetiology” tumours. Adapted from [4]

There is accumulating evidence of synergistic effects on hepatocarcinogenesis between MAFLD and other aetiologies of liver disease such as HBV, HCV and ARLD. Hence, the paradigm of attributing HCC to a single aetiology likely underestimates the true impact of MAFLD on HCC development. Several studies have quantified the size of this combined MAFLD aetiology subgroup and suggests that it is substantial (Fig. 3). The ITA.LI.CA registry estimated mixed-MAFLD tumours to constitute 48.4% of new HCC diagnoses in 2018–2019, of which HCV was the most common cofactor (67%). The proportion of mixed MAFLD HCC had remained fairly stable since 2002–2003, likely reflective of a simultaneous increasing prevalence of MAFLD offset by decreasing prevalence of viral aetiologies [31]. Similarly, the Geneva Cancer Registry study estimated the size of this combined MAFLD aetiology group to be 41% with HCV and ARLD being the most common cofactors [32].

Although the prevalence of MAFLD in HCV infection has not been well defined, evidence does suggest that amongst patients with HCV, both steatosis and metabolic dysfunction are highly prevalent, underdiagnosed and likely play a significant role in the divergent outcomes post-SVR on progressive liver dysfunction and HCC risk. For example, NHANES III data suggests patients with HCV are disproportionately affected by metabolic dysfunction with 69.6% of individuals with HCV having at least 1 major metabolic comorbidity, including 18.9% having T2DM and 20.9% having obesity [43]. In a cohort of 2611 Italian patients with advanced liver fibrosis or cirrhosis post-SVR, 58% of were reported to have metabolic dysfunction as defined using MAFLD criteria. Furthermore, metabolic dysfunction portended an increased risk of *de-novo* HCC (HR 1.97 95% CI 1.27–3.04), whereas steatosis visible on ultrasound did not predict HCC [44]. A Chinese study reported that steatosis (HR 2.4) and T2DM (HR 4.2) were both highly associated with HCC development post-SVR in a cohort of 1336 patients followed up post-SVR from either pegylated-interferon plus ribavirin or direct-acting antiviral (DAAs) therapy [45]. Another study found T2DM was independently associated with HCC development in 1000 patients post-SVR by 2.4 fold [46]. A study on 29,887 DAA treated US Veterans who achieved SVR reported that T2DM was independently associated with HCC, as well as cirrhosis and all-cause mortality (HR 1.32, 1.31, 1.25, respectively) [47]. MAFLD, therefore, may have a role in HCC risk stratification post-SVR, particularly amongst patients with F3 fibrosis in whom the need for surveillance is controversial. A recent meta-analysis estimated the pooled incidence of HCC development post-SVR amongst patients with cirrhosis and F3 fibrosis to be 2.1 and 0.5 per 100 patient years, respectively [48]. Application of MAFLD criteria has been proposed as one strategy which may be useful to inform surveillance strategies [49], however, studies to assess HCC incidence rates post-SVR in MAFLD vs non-MAFLD cohorts, both in cirrhosis and F3 fibrosis are waiting to be done.

The relationship between HBV infection and MAFLD is complex and incompletely understood. Lower prevalence of hepatic steatosis amongst HBV surface antigen (HBsAg)-positive patients compared to HBsAg-negative patients has been reported [50]. However, unsurprisingly given its high global prevalence in the general population, hepatic steatosis remains common in patients with HBV; a recent meta-analysis reported a prevalence of 34.93% [51]. In addition, the proportion of patients with HBV with superimposed MAFLD is increasing. One study from the Netherlands reported that patients with HBV referred to their centre after 2010 tended to have less active HBV-related disease including less e-antigen positivity, less indication for antiviral therapy and less severe fibrosis (OR 0.32, 0.30, 0.18, respectively) compared to patients referred prior to 2000. However, improvement in these metrics related to HBV was offset by higher prevalence of metabolic syndrome, steatosis and MAFLD (OR 2.77, 1.56, 1.35, respectively) (this does raise the question of whether the primary cause of liver disease and reason for referral for a subset of these “HBV cohorts” is in fact MAFLD) [52]. These findings are consistent with multiple other reports showing a temporal trend of worsening metabolic comorbidity and steatosis amongst HBV-infected individuals [53–55]. Interestingly, steatosis itself has been reported to be inversely associated with HBV viraemia and intrahepatic HBsAg expression [56, 57], however, despite this, MAFLD (with its associated metabolic dysfunction as opposed to simple steatosis) does appear to be associated with liver-related events including HCC amongst patients with HBV, highlighting the prognostic relevance of metabolic dysfunction. One study which used MAFLD criteria to stratify patients with HBV found that in a cohort of 1076 patients with HBV who underwent liver biopsy, the presence of MAFLD was associated with reduced event-free survival (using a composite endpoint of HCC, liver decompensation, liver transplantation, and all-cause mortality), while fatty liver disease without metabolic dysfunction (NAFLD-only), was not associated with adverse outcomes [58].

Regarding HCC risk specifically, simultaneous HBV and MAFLD appears to be common amongst HBV patients with HCC **(**Table 1**)**. One large Taiwanese study reporting that amongst a cohort of 800 patients diagnosed with early-stage HBV HCC between 2009 and 2018, 45.6% had concurrent MAFLD [59], while MAFLD prevalence in a Chinese cohort of 453 patients with HBV-related HCC was 57% [60]. A large Korean insurance registry study in HBsAg-positive individuals reported that the co-presence of MAFLD significantly increased the risk of HCC development with an adjusted hazard ratio of 1.37 [61]. MAFLD with T2DM was noted to have the highest risk for HCC development, however, all MAFLD sub-phenotypes had increased risk. Similarly, a Chinese study found metabolic syndrome to be independently associated with a twofold increased HCC risk amongst 6,545 prospectively enrolled individuals with HBV after adjusting for age, gender, cigarette smoking, alcohol consumption, liver cirrhosis, and elevated aspartate aminotransferase levels [62]. Some studies have reported poorer prognosis in patients with concurrent HBV MAFLD HCC, including increased HCC recurrence and all-cause mortality following surgical resection, as well as higher risk of death and progression [60, 63].

**Table 1:** Selected studies reporting the prevalence of MAFLD in cohorts of HCV, HBV, and ARLD-related HCC [32, 75–79]

Study	Study period	Population	HCC aetiology	MAFLD co-prevalence
Myers (2021)	1990–2014	*N* = 191 Geneva cancer registry (Switzerland)	HCV	43%
Conci (2022)	2008–2018	*N* = 1161 Italian He.RC.O.Le.S. Group registry, patients undergoing hepatectomy with HCC (Italy)	HCV	33%
Kim (2022)	2009–2022	*N* = 3732 HCC cases, derived from cohort of 63,233 who underwent health examination in 2009 with viral hepatitis [74](Republic of Korea)	HBV + HCV	18%
Lin (2022)	2009–2018	*N* = 800 Chang Gung Research Database (CGRD), BCLC 0/A HCC undergoing hepatectomy (Taiwan)	HBV	46%
Xue (2022)	2010–2020	*N* = 549 patients undergoing resection at single centre (China)	HBV	31%
Conci (2022)	2008–2018	*N* = 451 Italian He.RC.O.Le.S. Group registry, patients undergoing hepatectomy with HCC (Italy)	HBV	39%
Myers (2021)	1990–2014	*N* = 397 Geneva cancer registry (Switzerland)	ARLD	55%
Vanlerberghe (2022)	1990–2020	*N* = 123 Transplanted for ARLD-related HCC at single centre (Belgium)	ARLD	80%
Reggidori (2023)	2006–2020	*N* = 1391 ITA.LI.CA registry, diagnosed with ARLD-related HCC between (Italy)	ARLD	80%

There is a paucity of epidemiological and outcomes data on combined ARLD and MAFLD-related HCC, however, alcohol is known to have a synergistic effect with T2DM and obesity on the progression of liver fibrosis and development of HCC [64]. A prospective population-based study of 23,712 Taiwanese residents found that alcohol use (defined as use of any quantity greater than 4 days per week for 1 year) and obesity (BMI > 30 kg/m2) were significantly and synergistically associated with HCC (HR 7.19) [65]. A French study reported that in a cohort of 478 biopsy proven patients with cirrhosis from ARLD, overweight (BMI > 25 kg/m2) or obesity (BMI > 30 kg/m2) and T2DM were both independent predictors of HCC development (HR 2.0, 2.8, 1.4, respectively) [66]. Obesity was also an independent predictor of HCC development in patients with cirrhosis due to ARLD in an analysis of the United Network for Organ Sharing database (aHR 3.2) [67]. Several studies have reported an extremely high prevalence of MAFLD amongst individuals with ARLD-related HCC. In the ITA.LI.CA cohort, 80% of 1391 ARLD-related HCC cases between 2006 and 2020 were reported to have MAFLD [68], while a Belgian cohort of 142 ARLD-related HCC individuals who underwent transplant between 1990 and 2020 similarly had a MAFLD prevalence of 79.5% [69]. Taken together, these results suggest that MAFLD is not only very common, but also has synergistic effects with ARLD for the development of HCC, although further studies to quantify the strength of this relationship as well as outcomes are still needed.

Epidemiological research assessing alcohol consumption is challenging, with studies often plagued by methodological issues including failure to account for the pattern and type of alcohol consumption, changing habits over time, issues with under reporting, and incomplete adjustment for confounders. An Austrian study found that amongst 114 patients from outpatient liver clinics with presumed fatty liver disease, 29.8% were found to have evidence of moderate to excessive alcohol consumption on hair ethylglucuronide testing [70]. Furthermore, those with biopsy confirmed metabolic steatohepatitis (MeSH) are known to have altered gut microflora with an increased abundance of alcohol producing bacteria, with blood-ethanol concentrations that are higher than healthy controls or obese patients without liver disease [71]. These studies highlight that even in “bona fide MAFLD” cohorts, disentangling the effects of MAFLD and alcohol has significant challenges. In contrast, one study found that 68.7% of patients undergoing transplant for ARLD had concomitant MAFLD [69], while patients who have undergone liver transplant for ARLD-related cirrhosis have the highest rates of de-novo steatosis (37% vs 26%), even in the setting of alcohol abstinence, suggesting other factors such as metabolic dysfunction predispose many of these patients to liver disease in the first place [72, 73]. Dichotomising liver disease or HCC aetiology into ARLD or MAFLD based off an arbitrary alcohol consumption cut-off of 20 g/day for women and 30 g/day ignores the continuum on which these two interacting disorders exist. Hopefully, the concept of MAFLD which is not mutually exclusive to ARLD will lead to wider recognition that many patients have multiple liver disease which each require appropriate diagnosis and management, while paving the way for further research in this area.

## MAFLD HCC mechanisms

### Excess weight, insulin resistance, lipotoxicity, oxidative stress

**Fig. 4:**
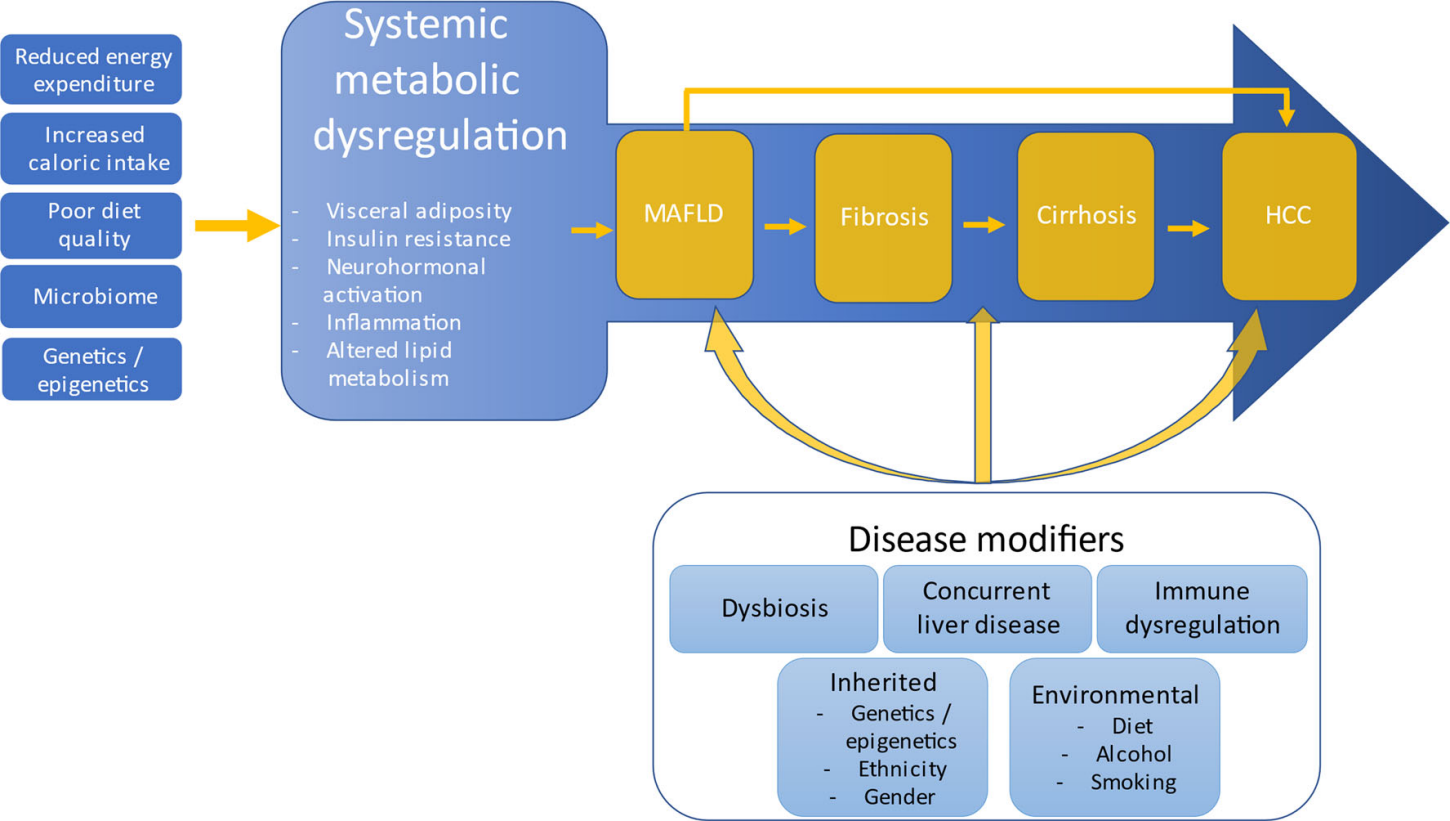
MAFLD-HCC mechanisms schematic. Systemic metabolic dysregulation crucially underpins MAFLD. Many interacting disease modifiers influence the phenotypic manifestations and progression to cirrhosis and/or HCC

The pathway from metabolic dysfunction to HCC is complex and multifactorial **(**Fig. 4**)**. Excess weight and ensuing insulin resistance is crucially linked to the development of hepatic steatosis via several mechanisms including increased release of non-esterified fatty acids (NEFA) from adipocytes and their delivery to hepatocytes, as well as increased *de-novo* lipogenesis (DNL) from carbohydrates in the liver. Upregulated DNL is a crucial feature of MAFLD and HCC development, with ubiquitin-specific protease 22 (USP22) recently identified as a key regulator of DNL in MAFLD HCC, with high USP22 expression associated with poor prognosis and overall survival [80]. Accumulation of toxic lipid species can cause injury and cell death in hepatocytes and non-parenchymal liver cells via generation of reactive oxygen species (ROS), oxidative stress, endoplasmic reticulum stress and inflammasome activation. Oxidative stress-induced DNA damage can predispose to carcinogenesis [81]. Increasing mutational burden in genes regulating lipid processing and storage *FOXO1, CIDEB and GPAM* develop due to the selective pressure on hepatocytes induced by lipotoxicity. These mutations may protect hepatocytes from lipotoxicity but also predispose to malignancy [82]. The oxidative environment generated in MAFLD can also lead to oxidation and inactivation of protein tyrosine phosphatases (PTPs), leading to increased STAT-1 and STAT-3 signalling. Interestingly STAT-1 signalling appears to mediate steatohepatitis and fibrosis but not carcinogenesis, while STAT-3 signalling increases carcinogenesis in the absence of liver damage, in keeping with the clinical observation that HCC and fibrosis can occur as independent events [83]. Insulin resistance induces compensatory hyperinsulinaemia which increases production of insulin-like growth factor 1 (IGF-1). This further promotes cellular proliferation and inhibits apoptosis [84]. Numerous hepatokines and adipokines are also implicated in hepatocarcinogeneisis [85]. Leptin is an important adipokine which decreases appetite and increases energy expenditure via its actions on the hypothalamus. Leptin is increased in obesity, MAFLD and HCC and acts as a mitogen which stimulates cellular proliferation and is associated with carcinogenesis in obesity [86, 87].

### Immune microenvironment

The immune microenvironment of MAFLD appears to be distinct compared to other chronic liver diseases, creative a permissive setting for the development of HCC. For example, dysregulated lipid metabolism causes accumulation of free fatty acids (in particular linoleic acid) leading to selective CD4 + loss. CD4 + cells are important in inhibiting HCC initiation and mediating tumour regression [88, 89]. T_H_17 cells also infiltrate the liver in response to hepatic DNA damage, triggering neutrophil recruitment via IL-17A, leading to subsequent fatty acid accumulation, steatohepatitis and HCC [90]. Liver resident macrophages (Kupffer cells) are another important regulator of inflammatory and fibrotic signalling cascades in MAFLD which may predispose to HCC [91]. Studies have recently highlighted the role for aberrant T cell activation in MAFLD and HCC. One study reported on an abundance of liver-resident CD8 + T cells in MAFLD mice with markers of exhaustion and effector functions. These cells were triggered to become “auto-aggressive” by IL-15-induced downregulation of the transcription factor FOXO1 followed by metabolic stimuli exposure including acetate [92]. Another study reported the presence CD8 + PD1 + T cells in MeSH with features of exhaustion, lacking in immune surveillance functions and with tissue damaging functions. Expansion of this population with immunotherapy led to increasing liver cancer incidence, highlighting their potential role in MAFLD HCC pathogenesis [93].

A confounder in all such studies is that patients are typically considered to have MAFLD-related or viral (or other) hepatitis-related HCC. This ignores the role and impact of concomitant MAFLD in patients with liver disease from another aetiology. Hence, we suggest that future studies on the immune microenvironment should focus on HCC development in MAFLD, in those with MAFLD and another liver disease and those with another liver disease but without concomitant MAFLD (Fig. 3). The mechanisms for HCC development, the outcomes, and response to therapy are likely very different among these groups. Such mechanistic insights are especially important in the era of immunotherapy in order to leverage the most effective therapies for patients.

### Genetics

Complex interactions between genes and the environment shape MAFLD phenotype and progression including towards HCC. The importance of genetics is illustrated by mono and dizygotic twin studies estimating that 61% of liver fat content is genetically determined [94]. A number of single nucleotide polymorphisms (SNPs) associated with abnormal hepatocyte lipid metabolism are linked to hepatic steatosis and increase HCC risk [95]. One of the most notable is the rs738409 SNP in *PNPLA3* that changes residue 148 of patatin-like phospholipase 3 (PNPLA3) from isoleucine to methionine (G allele), which causes impaired degradation of lipid droplets in hepatocytes. PNPLA3 rs738409 C > G is associated with increased risk of HCC in MAFLD with an approximate doubling of HCC risk for each copy of the minor (G) allele [96]. TM6SF2 SNP rs58542926 is associated with upregulation of cholesterol and fatty acid biosynthesis pathways and increases HCC risk relative to those without fatty liver without the variant (OR 1.92) [97, 98]. MBOAT7 rs641738 is also associated with increased intrahepatic triglyceride content and is independently associated with HCC (OR 2.10) [99]. A loss of function variant of *GCKR* rs1260326 (encoding glucokinase regulator) increases de novo lipogenesis and increases HCC relative to MeSH without the variant (OR 1.84) [100]. Combining all this data, a polygenic risk score (PRS) has been proposed as a tool to stratify MAFLD individuals at risk for HCC to improve surveillance yield [101].

### Microbiome

The gut microbiome is an important regulator of digestion and multiple metabolic processes, and influences the susceptibility to many liver diseases from steatosis to steatohepatitis, fibrosis and HCC. Dysbiosis is known to occur in MAFLD, with distinct but overlapping microbial signatures at the level of phylum, family and genera reported amongst MAFLD patients with simple steatosis, steatohepatitis and advanced fibrosis [102, 103]. Dysbiosis contributes to hepatic steatosis by increasing short-chain fatty acid (SCFA) generation which serves as a substrate for hepatic *de-novo* lipogenesis, as well as increasing absorption of monosaccharides across the intestine.

MAFLD is also characterised by increased intestinal permeability due to alterations in tight junctions which may lead to increased hepatic exposure to pro-inflammatory and oncogenic microbes and microbial products [104–106]. Increased translocation of microbial-associated molecular patterns (MAMPs) and danger-associated molecular patterns (DAMPs) can activate toll like receptors (TLRs) on Kupffer cells, hepatocytes and stellate cells, triggering inflammatory and fibrotic signalling cascades and predisposing to carcinogenesis [107].

The gut microbiome also has important immunomodulatory effects which can predispose to HCC. A recent study reported enrichment in SCFA producing bacteria in MAFLD-HCC patients which resulted in immunomodulation towards immunosuppression characterised by increased peripheral T_reg_ cells and reduced CD8 + T cells [108]. Bile acids are another important metabolite linking the microbiome to HCC development. Bile acids are regulators of lipid and glucose handling and modulate inflammation in MAFLD. Since the gut microbiome converts primary bile acids to secondary bile acids, it has a profound impact on bile acid signalling via suppression of farnesoid X receptor (FXR) signalling. This predisposes to liver damage [109], as well as increasing the levels of the secondary bile acid deoxycholic acid (DCA) (a gut bacterial metabolite known to cause DNA damage) [110].

### Chemoprevention

Addressing well-established lifestyle-related modifiable risk factors remain a key tool in HCC prevention in individuals with MAFLD. These include improving dietary patterns (including a hypocaloric and Mediterranean diet), increasing physical activity, measures to achieve weight loss and avoidance of other carcinogens including smoking and alcohol [111]. Indeed, mounting evidence implicates even moderate quantities of alcohol as a cofactor for HCC development in MAFLD [112]. A number of other pharmacotherapies have garnered interest as chemopreventative agents, including coffee, metformin, aspirin, statins and several novel T2DM therapies [113] **(**Table 2**)**. Chemoprevention is yet another unmet need and a number of these therapies show promise, however, in the absence of prospective efficacy data, coffee is the only chemopreventative therapy to be explicitly endorsed by any of the major society guidelines[114], highlighting the need for dedicated prospective studies.

**Table 2:** Chemopreventative therapies investigated for MAFLD-related HCC

Therapy	Evidence	Proposed mechanisms
Metformin	Multiple large retrospective observational studies reporting reduced incidence by ~ 50%. No benefit seen in post hoc analysis of two randomised control trials [115, 116]	Induced AMPK activation, autophagy activation, reduced hypoxia‐induced HIF‐1α accumulation [115]
Aspirin	Multiple large national cohort studies showing reduced HCC incidence. No HCC specific randomised control trial data [117, 118]	COX-2 overexpressed in HCC and hepatic stellate cells, mediates profibrotic and proliferative signalling cascades including protein kinase 3, mammalian target of rapamycin, and nuclear factor κB pathways [119]
Statins	Reduced HCC risk reported in multiple large retrospective observational studies. Few specifically investigating MAFLD-HCC. No benefit in post hoc analysis of randomised control trials [119, 120]. Lipophilic statins may be more efficacious than hydrophilic statins [121]	Pleotropic anti-cancer effects. Block oncogenic pathways including Ras-MAPK and PI3K/Akt. Inhibit activation of proteasome pathways. Block Myc phosphorylation. Anti-angiogenic effects. Reduce inflammation and fibrosis pathways [119]
SGLT2 inhibitors	Pre-clinical studies show inhibitory effect of canagliflozin effect on HCC cell lines in vitro. Reduced HCC in Western diet-fed melanocortin 4 receptor deficient mice [122–124]	Inhibits SGLT2-dependent glucose uptake on HCC cells, induces G2/M arrest, facilitates apoptosis via suppression of AKT phosphorylation [122–124]
Angiotensin converting enzyme inhibitors (ACEIs)	Retrospective territory wide cohort study of 12,327 MAFLD patients in Hong Kong showed ACEI associated with reduced HCC risk after propensity score weighting (HR 0.46). Other retrospective studies with conflicting results Protective effect on recurrence post-curative HCC treatment when given in combination with vitamin K2 or branched chain amino acids [125–128]	Inhibition of angiogenesis and tumour cell proliferation, suppression of inflammation and reduction in inflammatory cytokines, pro-apoptotic effects [125]
Coffee	Meta-analysis of 15 prospective cohort studies, dose-dependent reduction in HCC in high vs no/occasional coffee drinkers (RR 0.50) [129]	Multiple biologically active components including phenolic compounds and diterpenes with antioxidant properties [130]
Thiazolidinediones	Several large retrospective and meta-analysis studies with conflicting results. Recent meta-analysis (280,567 participants, 19,242 HCC cases) reporting reduced HCC risk (aOR = 0.92) [113, 131]	Insulin sensitisation, enhanced glucose metabolism, promote cell cycle arrest, induce apoptosis, inhibit cell invasion, stimulation of anti-angiogenic and pro-differentiation pathways, reduced stellate cell activation [113, 132, 133]
DPP-4 inhibitors	Retrospective cohort study from Taiwan reporting reduced HCC incidence with DPP-4 inhibitor use in individuals with HCV and T2DM (aHR 0.59). Pre-clinical data showing reduced hepatocarcinogenesis with DPP-4 inhibitors [134–136]	Suppression of pro-inflammatory/profibrotic macrophage phenotype. Activation of NK and T cell chemotaxis into HCC. Suppression of tumour angiogenesis [135–137]
GLP-1 receptor agonists	Reduced rates of hepatic decompensation with GLP-1 agonist compared to other diabetic medications in cirrhotic patients with T2DM. Minimal clinical data on HCC incidence. Pre-clinical studies reporting GLP-1 agonists promote apoptosis of hepatoma cells in vitro, and suppress carcinogenesis in MAFLD mouse models [138–140]	Amelioration of hyperglycaemia and obesity. Inhibition of multiple oncogenic signalling pathways including EGFR/STAT3 and JNK [139–141]

## MAFLD HCC clinical presentation

MAFLD-related HCC appears to exhibit a distinct phenotype in terms of both patient and tumour characteristics. A recent meta-analysis which included 61 studies on MAFLD-HCC reported that these patients were older (mean difference 5·62 years), with a higher mean BMI (mean difference 2·99 kg/m^2^), and more likely to have metabolic complications including diabetes (OR 4·31), hypertension (OR 2·84), dyslipidaemia (OR 3·43) and cardiovascular disease (OR 2·23) compared to HCC due to other aetiologies [142]. In addition, MAFLD HCC tumours tend to be larger (mean difference 0·67 cm), are more likely to be uninodular (OR 1·36) and occur on a background of non-cirrhotic liver disease (38.5% vs 21.7% for HBV, 6.4% for HCV and 9.1% for ARLD). Importantly, only 32.8% of patients with MAFLD HCC were undergoing surveillance compared to 55.7% of patients with other aetiologies, reflecting the fact that a significant proportion patients would not have had indications for routine surveillance based on current recommendations.

Several studies have examined the clinical characteristics of patients with HCC with MAFLD, utilising the newer disease definition and including the non-overlap group (MAFLD without NAFLD). An analysis of the large Italian ITA.LI.CA HCC registry classified tumours into either single aetiology MAFLD (S-MAFLD), mixed-aetiology MAFLD (M-MAFLD) or non-MAFLD, and found that S-MAFLD tumours were larger, more frequently associated with extrahepatic metastases, but less frequent clinically relevant portal hypertension or MELD score > 10 and with lower AFP compared with non-MAFLD tumours. Interestingly the M-MAFLD group, appeared to have a distinct clinical phenotype. Compared to non-MAFLD tumours, M-MAFLD tended to occur on less advanced cirrhosis by MELD score > 10 or significant portal hypertension, as well as being older, having poorer performance status (ECOG > 0) and lower AFP. However, this subgroup was also distinct from S-MAFLD tumours due to being more likely to have cirrhosis, with smaller tumours and higher AFP level [31]. Another study from Switzerland compared the non-overlapping MAFLD group (i.e., MAFLD without NAFLD) to NAFLD HCC, and found MAFLD HCC tended to occur in settings of more severe liver dysfunction, more severe portal hypertension and were less likely to receive curative therapy [32].

## Outcomes

### Overall survival

Overall mortality for MAFLD HCC was reported by the ITA.LI.CA HCC registry study and reported a significantly longer median survival in patients with single aetiology MAFLD HCC (28.1 months) compared to non-MAFLD HCC (23.8 months) after adjusting for baseline differences between subgroups and lead time bias, as well as a lower competing risk of death related to HCC progression compared to non-MAFLD. This was partially offset by a significantly higher risk of death by other causes in single aetiology MAFLD. The authors postulated that these results hint towards a less biologically aggressive phenotype of MAFLD HCC, particularly because MAFLD HCC tend to be more advanced at time of diagnosis [31]. MAFLD HCC patients were also more likely to be treated by resection, but less likely to receive liver transplant.

Other studies assessing overall survival in MAFLD HCC have documented conflicting results, including a retrospective cohort study of 1119 HCC patients in Germany which reported a shorter median overall survival for MAFLD HCC compared to non-MAFLD (11.28 vs 15.5 months). Higher BMI was associated with longer survival in all HCC groups. There was a trend towards more advanced HCC at diagnosis in the MAFLD group (trend towards larger tumour size, more multifocal disease and distant metastases), thus differences in surveillance and lead time bias may explain some of these results [143]. Of note, a recent meta-analysis reported no difference in overall survival between MAFLD and non-MAFLD HCC, however, MAFLD HCC was associated with improved disease-free survival including amongst those who received curative therapy. When the analysis was limited only to patients with cirrhosis, however, MAFLD HCC was associated with worse overall survival. MAFLD HCC was associated with a similar overall likelihood of receiving curative therapy, with a higher likelihood of resection but a lower likelihood of receiving transplant. There was substantial heterogeneity between studies [142]. Another study found no difference in overall survival between cirrhotic MAFLD HCC and non-cirrhotic MAFLD HCC as cirrhotic patients were more likely to have their tumours detected on surveillance imaging and therefore were found at an earlier stage, thereby offsetting the detrimental effect of their more advanced liver dysfunction and thus highlighting the importance of effective surveillance on outcomes [144].

### Outcomes post-curative therapy

Outcomes post-MAFLD-HCC resection were recently evaluated in a large Italian cohort who underwent HCC resection. Consistent with other studies, MAFLD HCC tended to occur in older patients with more metabolic comorbidity, with larger tumours and lower rates of cirrhosis. MALFD HCC was found to have the lowest overall survival compared to HCV, HBV and ARLD post resection, and was an independent prognostic factor on multivariable survival analysis. Recurrence free survival was similar. The short-term (90 day) post-operative mortality rate was nearly double that of other aetiologies at 5.9% despite similar rates of major complications and post-hepatectomy liver failure (PHLF) [75]. In contrast, a recent meta-analysis reported a superior overall and disease free survival of MAFLD HCC resections compared to other aetiologies [145], suggesting longer term outcomes may in fact be favourable in well-selected patients despite the short-term perioperative cardiometabolic risk.

Amongst patients who receive a liver transplant for HCC, MAFLD is an increasingly common cause of underlying liver disease, increasing from 1.3% in 2002–2004 to 8.3% from 2014–2016 in a European registry study [146]. There were no significant differences in post liver transplant survival outcomes or graft survival outcomes between MAFLD and non-MAFLD recipients reported, either for recipients with or without HCC [146] although recurrence of MAFLD is known to be common post-transplant [147]. A United Network for Organ Sharing (UNOS) database did report superior overall survival in patients transplanted for MAFLD, however, in the sub-population who received transplant for HCC, there was no difference in overall survival by liver disease aetiology [148]. Post-transplant tumour recurrence between MAFLD and non-MAFLD also appears similar, although a longer time to recurrence (22.6 vs 13.3 months) has been reported in MAFLD [149].

### Outcomes post-systemic therapy

The treatment landscape for advanced HCC has changed dramatically and there is growing interest in the influence of liver disease aetiology on the efficacy of systemic therapy. Sorafenib was the first multikinase inhibitor (MKI) approved for first line treatment of advanced HCC in 2008 following the SHARP trial showed an improvement in median survival and time to progression [150], followed by the REFLECT trial which showed non-inferiority of the MKI lenvatinib in 2018 [151]. Immunotherapy subsequently emerged as first line therapy in advanced-stage HCC following the IMbrave150 trial which showed superior overall and progression free survival with anti-programmed cell death-ligand 1 (PD-L1) atezolizumab in combination with anti-vascular endothelial growth factor (VEGF) bevacizumb compared to sorafenib [152]. More recently, the HIMALAYA trial demonstrated the efficacy of a dual immunotherapy approach with combination anti-cytotoxic T lymphocyte–associated antigen 4 (CTLA-4) tremelimumab plus durvalumab (anti-PD-L1) with superior overall survival compared to sorafenib [153], establishing a new first line option. Another phase III study, COSMIC-312, compared atezolizumab in combination with the MKI cabozantinib to sorafenib and found an improvement in progression free survival but no overall survival benefit over sorafenib [154]. Interestingly, the results from these recent trials did suggest a differing efficacy of immunotherapy depending on the aetiology of liver disease. COSMIC-312 reported that overall survival appearing longer in the immunotherapy arm in patients with HBV (HR 0.53 95% CI 0.33–0.87) but not with non-viral aetiology (HR 1.18 95% CI 0.78–1.79). Likewise IMbrave150 reported a beneficial effect of immunotherapy in patients with HBV (HR 0.51 95% CI 0.32–0.81) and HCV (HR 0.43 95% CI 0.22–0.87) but not in non-viral HCC (0.91 95% CI 0.52–1.60).

Pfister et al. proposed a mechanism for this observation, by reporting an increased population of hepatic CD8 + PD1 + T cells in MeSH with features of exhaustion, lacking in immune surveillance functions and with tissue damaging functions. In pre-clinical models, prophylactic treatment with anti-PD-1 therapy expanded this CD8 + PD1 + population and led to an increase in HCC, while anti-PD-1 treatment in MeSH-HCC pre-clinical models also expanded this population in tumours but without tumour regression.

The investigators performed a meta-analysis of three large phase III RCTs which reported overall survival data for immunotherapy for advanced HCC (IMbrave150, KEYNOTE-240 and CheckMate-459) and reported superior survival of immunotherapy compared to the control arm overall (HR 0.77, 95% CI 0.63–0.94), and in subgroups with HBV-related HCC (*n* = 574, *P* = 0.0008) and HCV-related HCC (*n* = 345, *P* = 0.04), but not in patients with non-viral HCC (*n* = 737, *P* = 0.39) [93]. A subsequent meta-analysis of five RCTs (IMbrave150, COSMIC-312, CheckMate 459, KEYNOTE-240 and HIMALAYA) also concluded that viral HCC responds better to immunotherapy compared to non-viral aetiology (P* = 0.0469) [95].

Real world data from a recent retrospective analysis of prospectively collected data from Italy, Japan, Republic of Korea and UK likewise reported that lenvatinib was associated with superior overall survival (aHR 0.65 95% CI 0.44–0.95) and progression free survival (aHR 0.67 95% CI 0.51–0.86) compared to atezolizumab and bevacizumab in advanced HCC, which was driven by superior overall survival in patients with MAFLD (HR 0.46 0.26–0.84) and MeSH (HR 0.55 95% CI 0.38–0.82) [155]. The results were consistent following a propensity matched analysis. Similarly, an international study from Japan and Italy reported that in a cohort of 320 patients with advanced HCC treated with lenvatinib, MAFLD aetiology was associated with significantly longer overall survival (median 21.1 vs 15.1 months). It is difficult to conclude whether this was due to a differential response to lenvatinib or to other between-group differences such as treatment duration, liver function or comorbidities [156]. Caution is necessary when drawing conclusions from retrospective analyses. Furthermore, it should be emphasised that the RCTs tend to report data from a “non-viral HCC” subgroup which includes both MAFLD, ARLD and potentially other rarer liver diseases. Also of note, the HIMALAYA trial did report improved overall survival in the immunotherapy arm for non-viral HCC (HR 0.74 95% CI 0.57–0.95) [153]. Currently, none of the major society guidelines recommend any significant differences in the management of MAFLD vs non-MAFLD HCC [114, 157, 158], however, these results highlight the need for well-designed prospective studies to determine the clinical impact of underlying aetiology on responsiveness to treatment. Indeed, in the era of precision medicine, other biomarkers as predictors of disease response beyond liver disease aetiology are lacking and desperately needed.

## MAFLD HCC screening

Surveillance is recommended in patients at high risk of HCC due to the differential prognosis based on HCC stage at time of diagnosis. A 2022 meta-analysis of 59 studies reported that screening was associated with earlier stage detection (RR 1.86, 95% CI 1.73–1.98) and improved overall survival (HR 0.64, 95% CI 0.59–0.69) in patients with cirrhosis [159]. Screening is generally considered to be cost effective in those with an estimated yearly incidence of 1–1.5% [114], albeit this is context and country cost dependent. Thus, despite there being no studies specifically examining screening in cirrhotic MAFLD populations, it is widely recommended that these patients undergo screening given a reported HCC incidence of 0.9 – 2.6% [2, 23, 24, 114, 157, 158].

It is well recognised that a significant proportion of HCC in patients with MAFLD occurs in the absence of cirrhosis, estimated to be 38% in a 2018 meta-analysis [25]. This is a function of the high global prevalence of non-cirrhotic MAFLD. There is a substantially reduced annual HCC risk amongst non-cirrhotic individuals and routine screening in this population is not recommended [160]. There is also limited data regarding the benefit of screening of patients with F3 fibrosis. Of note, a Veterans Health Administration cohort study found an annual HCC incidence of > 1% in individuals with a FIB-4 score > 2.67, irrespective of a known cirrhosis diagnosis, suggesting this to be a population that may benefit from screening [26]. There is a need therefore for validated risk stratification models to identify non-cirrhotic patients with MAFLD who will benefit from screening, given that current screening algorithms will continue to result in a high proportion of MAFLD HCC detected outside of routine surveillance.

Society guidelines currently recommend 6 monthly transabdominal ultrasound surveillance with or without serum AFP measurement as a surveillance strategy [114, 157, 158]. However, the inherent limitations of ultrasound in terms of its sensitivity and operator dependency are amplified in patients with MAFLD. A meta-analysis of 32 studies reported a sensitivity of ultrasound of only 47% for early-stage HCC [161]. Furthermore, a retrospective cohort study on 941 patients undergoing regular surveillance for HCC found that 20.3% of ultrasound examinations were inadequate to exclude HCC, with body mass index category (OR 1.67) and MAFLD aetiology (OR 2.87) both independent predictors of an inadequate examination [162]. Computed tomography (CT) imaging as an alternative is limited primarily by increased cost, risk of contrast-related complications and requirement for repeated exposures to ionising radiation. Magnetic resonance imaging (MRI) with liver specific contrast has similarly been shown to have improved sensitivity for very early-stage HCC compared to ultrasound (84.8% vs 27.3%) with a lower false positive rate in patients with advanced cirrhosis at high risk of HCC [163]. However, this strategy may not be cost effective or feasible in most resource limited settings. The use of abbreviated non-contrast MRI (NC-MRI) protocols may be advantageous over conventional MRI protocols in terms of time and cost, with a typical scan time of 15–20 min compared to 40–45 min for conventional MRI protocol. A meta-analysis reported a pooled sensitivity of 77.1% for lesions < 2 cm with NC-MRI which compares favourably to 47% reported by a previous meta-analysis of US [161, 164], thus NC-MRI as a surveillance tool appears promising.

Use of serological biomarker panels are another surveillance strategy to overcome the current limitations, of which GALAD (combining gender, age, AFP, AFP-L3%, and DCP) is perhaps the most mature in terms of validation. A case–control study of 125 patients in Germany with HCC due to MeSH showed that GALAD had a sensitivity of 68% and specificity of 95% with AUC of 0.91 for detecting HCC within Milan criteria [165]. However, these results require further validation. Liquid biopsy techniques, often focussing on DNA methylation panels arising from circulating tumour cells have also been studied and show promising results [166], likewise identification of circulating lipid metabolite signatures to identify MAFLD HCC may be another promising strategy [167]. Improved surveillance methods are a major unmet need to improve the dismal proportion (32%) of MAFLD HCC detected on surveillance [142].

## Conclusion

The obesity epidemic has resulted in the HCC landscape evolving from one in which HCC is concentrated amongst high-risk populations with easily identifiable risk factors, to one of increasing prevalence amongst “low risk” populations. MAFLD HCC presents unique challenges in terms of identifying at risk populations, surveillance, as well as management of HCC, their underlying liver disease and comorbidities. Furthermore, the increasing prevalence of MAFLD amongst patients with other liver diseases necessitates a more holistic approach to identifying and managing multiple concurrent interacting liver diseases. Hopefully, the MAFLD framework will facilitate this paradigm shift moving forward.
